# On the Theory and Practice of Midwifery

**Published:** 1861-01

**Authors:** 


ART. III.—*On the Theory and Practice of Midwifery.* By FLEETWOOD CHURCHILL, M.D., M.R.I.A., Fellow of, and Professor of Midwifery and Diseases of Women and Children in the King and Queen's University in Ireland, etc., etc., etc. With additions by D. FRANCIS CONDIE, M.D., etc., etc. With one hundred and ninety-four illustrations. A new American, from the fourth corrected and enlarged English edition. 8vo., pp. 655. Philadelphia: Blanchard & Lea, 1860.

IT is not our intention to enter upon a formal review of the treatise of Dr. Churchill. The work has been too long before the medical public to render it necessary that we should at this time attempt any labored exposition of its claims to the character of a true and faithful exponent of the theory and practice of midwifery. The unanimous verdict of the profession in this country and abroad has been rendered in its favor; and it has been assigned a high rank among our standard systematic treatises, adapted alike for the use of the student and the practitioner of obstetrics.

Nothing more would seem to be demanded of us than to announce the appearance of a fourth edition and its American reprint, and point out some of its claims to our special notice.

In the present edition, Dr. Churchill, with that untiring industry so strikingly evinced in the preparation of all his works, has subjected every chapter to a thorough and careful revision, altering, amending, and adding to each, so far as his own experience and that of others has shown him to be demanded, in order to render his teachings, throughout, a clear and correct reflection of the actual condition of midwifery as a science and as an art.

There would be no little injustice done were the treatise of Dr. Churchill to be viewed in no other light than as a mere compilation from the writings of others. The author, it is true, has made free use of the valuable materials furnished him by his predecessors and contemporaries, but in no instance has he adopted as true the observations of others, however high may be their standing; or as correct their deductions, without having first instituted a careful scrutiny into their faithfulness and validity. The work before us is not, therefore, to be received simply as an exponent of what "the masters in obstetrics" have taught, and still continue to teach as true in theory and proper in practice, but as presenting, in addition to this, the evidence of the author's own experience and investigation in proof of the correctness of the teaching of others. It presents, consequently, a legitimate claim to the character of an original and safe guide to the learner, and, at the same time, an authoritative book of reference for the practitioner in his hour of need. It may be more safely recommended in both capacities from the perfect honesty with which its author has, in every instance, treated the opinions and contributions of his predecessors and contemporaries, the modesty with which his own views and observations are brought forward, and the perfect candor with which he invariably acknowledges a change in his opinions, whether in reference to a question of doctrine or of practice, when such change has been shown by sufficient evidence to bring him in closer relationship with the actual and the true.

In no portion of the treatise is this readiness of Dr. Churchill to go without hesitation wherever truth shall lead him more strikingly evinced than in the chapter on puerperal fever. The account it presents of the etiology, pathology, and treatment of that much-dreaded disease, it is true, is still somewhat confused and indefinite. When compared, however, with that given in the earlier editions, it will be found that the facts developed by the more intimate investigation to which, within a very recent period, every question connected with the nature and causation of child-bed fever has been subjected, by physicians enjoying the necessary opportunities, in different parts of the world, has produced an important modification of his views in respect to the character of one, at least, of the forms of the disease—its epidemic, or, as Dr. Churchill denominates it, its malignant form.

In reference to this, he very candidly acknowledges that "more extended experience has led him to doubt the accuracy" of the views of its pathology, taught by him in the earlier editions of the work under notice. He had in them adopted the views of those pathologists who refer puerperal fever invariably to a simple inflammation of the uterus and its appendages, regarding the general febrile phenomena merely as symptomatic of the local phlegmasia, whereas now he is convinced the disease "is something more than a local affection, and that the constitutional affection is often rather primary than secondary."

Nearly as important a change has taken place, also, in his views in respect to the etiology of puerperal fever. He fully recognizes its close relationship to epidemic erysipelas; its contagious character; the possibility of its being communicated from one puerperal female laboring under it, to another who is not, through the intermediacy of a third party apparently free from disease; and its intimate dependence upon a morbid condition of the blood, produced either by atmospheric malaria or contagious matter from without, or by the absorption of some noxious matter generated within the body of the patient.

We would call especial attention to the section which treats of the gastro-enteric form of fever incident to puerperal females. This affection, it is certain, cannot with propriety be considered as in any way related to true puerperal fever; still, we admit the propriety of treating of it in connection with the latter disease. It occasionally occurs as an epidemic, and in its early stages presents often a character of apparently great severity. From these circumstances it is liable, when it makes its appearance, to occasion a very great degree of anxiety, and to lead to the adoption of an active treatment by no means required in the management of a disease of comparatively only secondary importance. The account given by Dr. Churchill of its leading characteristics and usual course, though short, is replete with interest. We shall present, for the benefit of our readers, an outline sketch of it; inasmuch as the form of gastro-enteric fever referred to, while of much more frequent occurrence than is generally supposed, is but illy understood, and, in consequence, very apt to alarm and mislead the inexperienced practitioner. The description of the disease given by Dr. Churchill is the fullest and most accurate that we are acquainted with.

From his own observations during the epidemic prevalence of the disease in Dublin, in the years 1851 and 1852, and in isolated cases, he describes the attack as almost invariably occurring within one week after delivery—usually from the second to the fifth day. It is mostly marked by rigor, not very severe, however, but sufficiently well marked. In a few cases the rigor was repeated at the end of a week. The most striking symptoms of the attack are pain and diarrhœa. The pain comes on early—extending, at first, over the whole abdomen; being increased in paroxysms, and continuing until relieved by medicine. As the pain declines, it is experienced to a greater degree in one region—the left iliac—than in any other. After the first severe attack, frequent flatulent pains, with a great discharge of wind, are invariably complained of. There is, usually, tenderness of the abdomen proportionate in degree to the severity of the pain. When, however, equal and firm pressure is applied continuously, it gives relief instead of increasing the suffering. The tenderness is to the least extent over the uterine region. Diarrhœa is present in every case, even when the bowels, as is sometimes the case, are confined and loaded at the commencement of the attack, or when large doses of opium have been given for the relief of pain. The diarrhœa is unattended with either nausea or vomiting. In some cases the stools are few in number, but large in quantity, and unhealthy in appearance; in others they occur with great frequency and are followed by considerable exhaustion. The pulse is at first always frequent; from one hundred and twenty to one hundred and forty strokes in the minute. It gradually subsides as the distress of the patient diminishes. In a few instances, however, it has been known to continue of increased frequency for many days. There is, at first, increased heat of surface, but which soon subsides. There is, in general, a good deal of perspiration—occasionally it is excessive. Previously to the exhibition of opium, no thirst is complained of. The tongue is white, but not loaded or dry. The secretion of milk is very seldom disturbed. The lochia are, at first, generally diminished in quantity, or entirely suppressed, but return again uniformly in the course of a few hours. Occasionally they have a heavy smell for a day or two, at the end of which period they become natural and healthy.

As a general rule, the attack of the gastro-entric fever of child-bed lasts about a week. It is seldom that those attacked by it become convalescent at an earlier period. In a few instances the disease continues for a day or two longer.

Dr. Churchill believes the disease to be dependent upon severe irritation of the gastro-intestinal mucous membrane, of which the intense febrile excitement is symptomatic, the cause by which the intestinal irritation is produced being as yet unexplained. Occasionally, the womb and its appendages become involved, as indicated by the increase and permanency of the fever, perhaps its change of type; by the permanent suppression of the milk and lochia; and by the local tenderness on pressure over the uterine region. Such a complication places those cases in which it occurs in a very different category from those where the disease retains throughout its simple character, rendering, what is otherwise an affection of little danger, yielding readily to very simple treatment, one of more serious import, and which is not altogether safe from a fatal result.

It is only on its first onset that the disease just described is liable to be confounded with genuine puerperal fever. In those cases where the attack is attended by no complication, only a very few hours can elapse after its onset before its true character will be sufficiently revealed to obviate all unnecessary alarm.

The chapter on sudden death after delivery, which was introduced, we believe, for the first time, in the third edition, is replete with the most intense interest. It furnishes the best and fullest account we are acquainted with, in reference to the fearful accident of which it treats; and demands a careful study on the part of every one engaged in, or who is about to engage in the practice of obstetrics. Not that by the closest study of it he shall be able to learn the means to prevent, perhaps, in a solitary case, the occurrence of an event of which he is scarcely ever furnished with the slightest admonition—it being usually as unexpected as it is appalling. But with the hope that a close investigation of the causes by which it has been shown to be produced, and the circumstances under which it ordinarily occurs, may enable him to institute, at an early period, a judicious prophylactic course of treatment, in the hope that, in this manner, he may be spared the severe trial of beholding a young mother, whom he had but now congratulated upon her safe delivery, and fair prospect of a favorable getting up, become suddenly, and without any apparent cause, transformed into a lifeless corpse.

To the edition before us there is appended an essay on a subject which, though it never has been discussed before in any treatise on midwifery, is nevertheless of very great importance. The subject is "Obstetric morality"—or the right of the obstetrician to perform the operation of craniotomy whenever it becomes the sole means by which a chance is afforded for saving the life of the mother. The leading questions involved are discussed by Dr. Churchill with the utmost candor and ability. The circumstances under which, alone, the obstetrician may venture to destroy a living child, to insure the safety of the mother, without offending against morality or religion, are examined with care, and the legitimate conclusions deducible from an examination of all the considerations requisite to be taken into account in the formation of a correct judgment, are clearly announced.

Dr. Churchill is no advocate for the operation of craniotomy. His earnest desire is to restrict it within as narrow limits as possible compatible with a due regard for the greater value of the life of the mother in comparison with that of the unborn babe—which latter, we are to remember, must necessarily perish with her, in all those instances in which craniotomy is at all justifiable, should the operation not be resorted to. He urges the necessity of conducting, as far as possible, all cases of difficult, obstructed, or complicated labor, in such manner as shall most effectually conduce to the safety of both mother and child. Whenever it is possible, he would insist upon an early resort, in such cases, to the forceps; or when it has become evident, from the contracted size or deformity of her pelvis, that a female cannot be delivered at the full term of a living child, he suggests that the propriety of the induction, in her case, of premature labor, should be carefully weighed. He lays it down as a law of invariable application, "that craniotomy is never to be contemplated when a living child can, by any means compatible with the safety of the mother, be delivered *per vias naturales.*" He nevertheless shows most conclusively, that occasions do occur—happily, not very often—in which a resort to craniotomy is "not only necessary and justifiable, but imperatively demanded, even though the child be alive, if we would not voluntarily incur the responsibility of the mother's peril, and perhaps death."

No one can arise from the perusal of the essay under consideration, without a very full conviction of the importance of the subject discussed in it, the difficulties which surround it, and the very satisfactory manner in which it has been treated by the author. The substance of the essay first appeared in the *Dublin Quarterly Journal,* and was called forth by an article in the *Dublin Review,* in which the views of Dr. Churchill, in respect to craniotomy, as set forth in the earlier editions of his treatise on midwifery, are made the subject of animadversion, by one whom Dr. Churchill "strongly suspects is not a medical man."

In a second appendix are presented a series of very judicious remarks and instructions in reference to the qualifications and duties of "the monthly nurse." They are extracted, by consent of Dr. Churchill, from a little "manual for midwives and nurses" recently issued by him. The remarks and instructions are, confessedly, most excellent, and may, possibly, as the American editor supposes, "prove valuable to the young practitioner when called upon, for the first time, to take charge of the lying-in room." For ourselves, we believe that they would do far more good were they printed separately, in such a form as would permit of their being placed in the hands of the monthly nurse herself, for whose instruction alone they were expressly written. And provided, also, she could be induced to read, ponder, and inwardly digest them. They contain, certainly, the best summary we are acquainted with, of the ethics by which the nurse, in all her intercourse with her patient, and with the professional gentleman under whose charge the latter has placed herself, is to be governed.

With regard to the additions made by the American editor to the volume under notice, we have but little to say. We have no disposition whatever to dispute their fitness and general value. We would simply remark, that since the commencement of our career as medical journalists, we have protested against the practice pursued by many American physicians, of loaning their names as editors to foreign works on the various branches of the medical sciences. The practice, we are happy to say, is becoming every year less frequent, and we trust will before long entirely cease. We are convinced that to a certain extent it is a species of injustice on the part of the American bookseller, who thus appropriates for his exclusive profit the works of foreign authors, without any compensation to the latter; while, at the same time, it constitutes unquestionably an impediment to the increase and improvement of our national medical literature. It is proper, however, we should say in regard to the publication and editorship in this country of the work of Dr. Churchill now before us, as well as of his treatise on the diseases of females, that we are assured, from good authority, there has been an entire understanding with him; and that everything has been done in respect to both with his entire consent and approbation.

